# Sleep quality associate with the increased prevalence of cognitive impairment in coronary artery disease patients: A retrospective case–control study

**DOI:** 10.1515/med-2024-1034

**Published:** 2024-09-11

**Authors:** Min Liu, Jianning Ma, Kena Bao, Ye Gu, Jing Zhao, Dongmei Ren, Fang Zhu, Xiangdong Xu

**Affiliations:** Department of Scientific Research, Jiading District Central Hospital Affiliated Shanghai University of Medicine & Health Sciences, Shanghai, 201800, China; Department of Hospital Infection Control, The Fifth Affiliated Hospital, Sun Yat-sen University, Zhuhai, 519000, China; Department of Nursing, Jiading District Central Hospital Affiliated Shanghai University of Medicine & Health Sciences, Shanghai, 201800, China; Department of Cardiology, Jiading District Central Hospital Affiliated Shanghai University of Medicine & Health Sciences, Cheng Bei Road, Jiading District, Shanghai, 201800, China; Department of Nursing, Jiading District Central Hospital Affiliated Shanghai University of Medicine & Health Sciences, No. 1 Cheng Bei Road, Jiading District, Shanghai, 201800, China

**Keywords:** coronary artery disease, sleep quality, cognitive impairment, behavior pattern, risk factor

## Abstract

**Background:**

The pathogenesis of cognitive impairment (CI) in coronary artery disease (CAD) patients is still unclear and numerous influence factors could affect the CI status. The current studies suggest that sleep quality and behavior pattern are significant influence factors associated with CAD susceptibility.

**Methods:**

A total of 223 participants including 90 CAD patients with CI and 133 controls were enrolled into this retrospective study. Demographic information, laboratory test results, clinical diagnostic data, and questionnaire survey were collected to recognize the influencing factors of CI in CAD patients. Appropriate statistical methods are used to analyze these collected data.

**Results:**

Univariate analysis results of demographic information, laboratory test results, and questionnaire survey data revealed that the differences in fatigue symptom, age, HDL, TG, and sleep quality were statistically significant (*p* = 0.006, *p* = 0.000, *p* = 0.019, *p* = 0.028, and *p* = 0.037, respectively). Logistic regression analysis showed that age, fatigue, and sleep quality were the influence factors for CI in CAD population (*p* = 0.000, *p* = 0.035, and *p* = 0.017).

**Conclusions:**

Sleep quality, fatigue, and age were associated with the increased susceptibility of CI in CAD patients. Both CI state and its related factors were involved in the pathological process of CAD, these findings could offer additional information for the prevention and control of CAD.

## 1 Introduction

Coronary artery disease (CAD) is the leading cause of human mortality globally, especially among the elderly [1]. Due to chronic course, severe physical pain, and psychological suffering to the patients, the identification of risk factors is of clinical importance in this population [2,3]. Over the past few decades, hypertension, hyperlipidemia, smoking, and genetic variations were confirmed as the traditional risk factors for CAD; thus, controlling these risk factors would be beneficial to the disease management [4,5]. Currently, investigations have suggested that cognitive deficit was essential to CAD susceptibility [6]. CAD patients with cognitive impairment (CI) are more susceptible to poor outcomes and higher mortality rate, and CI may play an important role in the prognosis of cardiovascular disease [7–9]. Although cognitive problems and psychological suffering are increasingly prominent in clinical practice, very few studies focus on cognitive function and emotional disorders [10,11]. CI is a state of decline or impairment of cognitive function, and timely intervention can change the prognosis of the disease; therefore, more scholars pay attention to the early features of CI and progression of disease [12,13]. The current literature have confirmed that CI was also affected by many factors, such as sleep quality and behavior pattern [14–16]. Poor sleep quality could increase the susceptibility to CAD; meanwhile, CAD and its complications can also exacerbate sleep disorder. Sleep disorder is a modifiable CI risk factor, and both sleep quality and CI are linked to CAD [17,18]. Therefore, the investigation of CI status and identification of CI influencing factors in CAD patients would be beneficial for reducing the occurrence of adverse events.

## 2 Materials and methods

### 2.1 Study design

This study was a retrospective case–control study of patients with CAD who were hospitalized in cardiology department of Jiading District Central Hospital from Jan 2022 to Dec 2022.

### 2.2 Study population

All adult (≥18 years) patients who fulfill the inclusion criteria were admitted to the study, otherwise they were removed if they met the exclusion criteria. Finally, 223 participants were chosen as the research subjects of this study. Demographic information (including age, gender, family history, and the state of chronic disease) and laboratory test results were obtained from the electronic medical record system. Information about sleep quality and behavior characteristics was investigated by face-to-face interview survey.

### 2.3 Inclusion and exclusion criteria

#### 2.3.1 Inclusion criteria

(i) The diagnosis of CAD was confirmed by the gold standard coronary angiography (CAG).
(ii) Complete all the emotional assessment scales and obtain clear scores.
(iii) Adequate basic information and clinical findings were collected to analyze the distribution characters.

#### 2.3.2 Exclusion criteria

(i) Patients in coma or with mental disease were excluded from this research.
(ii) Patients with liver and kidney dysfunction, cardiopulmonary failure, and malignant tumor were ruled out.
(iii) Severely infected patients and immunocompromised patients were not allowed to participate in this study.
(iv) The CAD patients with pernicious anemia, malnutrition, hypothyroidism, and frailty syndrome were ruled out.
(v) Patients who did not wish to participate in this study or voluntarily withdrew from the study.

### 2.4 Grouping of participants

The CAD patients with cognitive disorder problems were classified as case group and the other patients without cognitive problems were brought into control group. After filtering, 90 patients were distributed into case group and the other 133 patients were distributed into control group.

### 2.5 Diagnostic criteria and definition

CAD: Meeting the diagnostic criteria for CAD in the *Guidelines for the Diagnosis* and *Treatment of Stable Coronary Artery Disease* and the diagnosis is confirmed by CAG.

Hypertension (HBP): Three consecutive non-same day blood pressure levels ≥140 mmHg systolic and/or ≥90 mmHg diastolic.

Sleep quality: Sleep quality is divided into good sleep quality, average sleep quality, and poor sleep quality according to the situation of sleep. The sleep time is 6–8 h, no difficulty falling asleep, many dreams, easy to wake up, and other conditions are defined as good sleep quality. Average sleep quality refers to the patients who can fall asleep, but slowly, occasionally wake up at night. The patients with poor sleep quality are those who have difficulty in falling asleep, easy to wake up, multiple dreams, early wake up or difficulty falling asleep after waking up, which appear more than 3 times a week.

Fatigue: The doctors make a comprehensive fatigue judgment by observing the patient’s clinical symptoms, medical history, and inquiry content. (1) Typical symptoms include dizziness, headache, weakness of the limbs, decreased energy, chest pain, nausea, vomiting, insomnia, memory loss, and difficulty concentrating. (2) In addition, doctors also pay attention to specific symptoms of the patient’s respiratory and circulatory system, such as dyspnea, lack of breath, chest tightness, shortness of breath, palpitations, discomfort in the precardiac area, bradycardia or tachycardia, and arrhythmias. (3) In the process of medical history inquiry and collection, find out whether the patient has a history of anemia, malnutrition, hypothyroidism, diabetes, or other chronic diseases. Combined with the above assessment information, if the patient has experienced a decrease in mobility almost every day for 2 weeks in the past few months, as well as a decrease in physical and psychological coping ability that requires increased rest for relief, then it can be judged as fatigue.

Smoking history: Those who continuously or cumulatively smoke for 6 months or more in their life are considered as smokers with a smoking history.

Drinking history: The patient who drank more than 40 g of liquor daily for more than 5 years or had a history of binge drinking within 2 weeks.

### 2.6 Assessment of CI

Mini mental status examination (MMSE) scale and the patients’ neurological symptoms (including reflexes, eye movements, walking, and balance) were combined to recognize the state of CI by the professional physician. Memory loss, language impairment, visuospatial impairment, executive impairment, numeracy impairment, and decreased ability of daily life were all taken into consideration in diagnosis of CI. Finally, if the MMSE scale score is greater than 27 points and no relevant clinical manifestation, then it is considered that the patient does not suffer from CI.

### 2.7 Biochemical examination

Blood samples collected from the enrolled patients were frozen at −70°C in refrigerator. Serum biochemical index, blood glucose data, items of myocardial infarction, and parameters of liver and kidney function were acquired and compared. Automatic biochemical analyzer (Abbott architect c16000, USA) was used to measure the levels of serum biochemicals and lipid metabolism indicators. HbA1c was tested by the method of high-performance liquid chromatography (HLC-723g7, Japan). Items of myocardial infarction were detected by automatic fluorescence immunoanalyzer (Radiometer, AQT90, Denmark).

### 2.8 Statistical method

All the collected data were analyzed using the software STATA version 12.0 (STATA Corp., College Station, TX). Measurement data were presented as mean ± standard deviation and the differences of these parameters were compared by the analysis of variance. The enumeration data and categorical variables were expressed as percentage, Chi-square analysis or Fisher’s exact test was used to compare the differences between them. Logistic regression analysis was used to identify the potential risk factors for CI. The *p* < 0.05 was considered as statistical association.


**Informed consent:** All the participants signed the informed consent.
**Ethical approval:** This study was approved by the Ethics Committee of Jiading District Central Hospital Affiliated Shanghai University of Medicine & Health Sciences (Approval number: No. 2022K29).

## 3 Results

### 3.1 Basic demographic information and clinical characteristics

**Figure 1:**
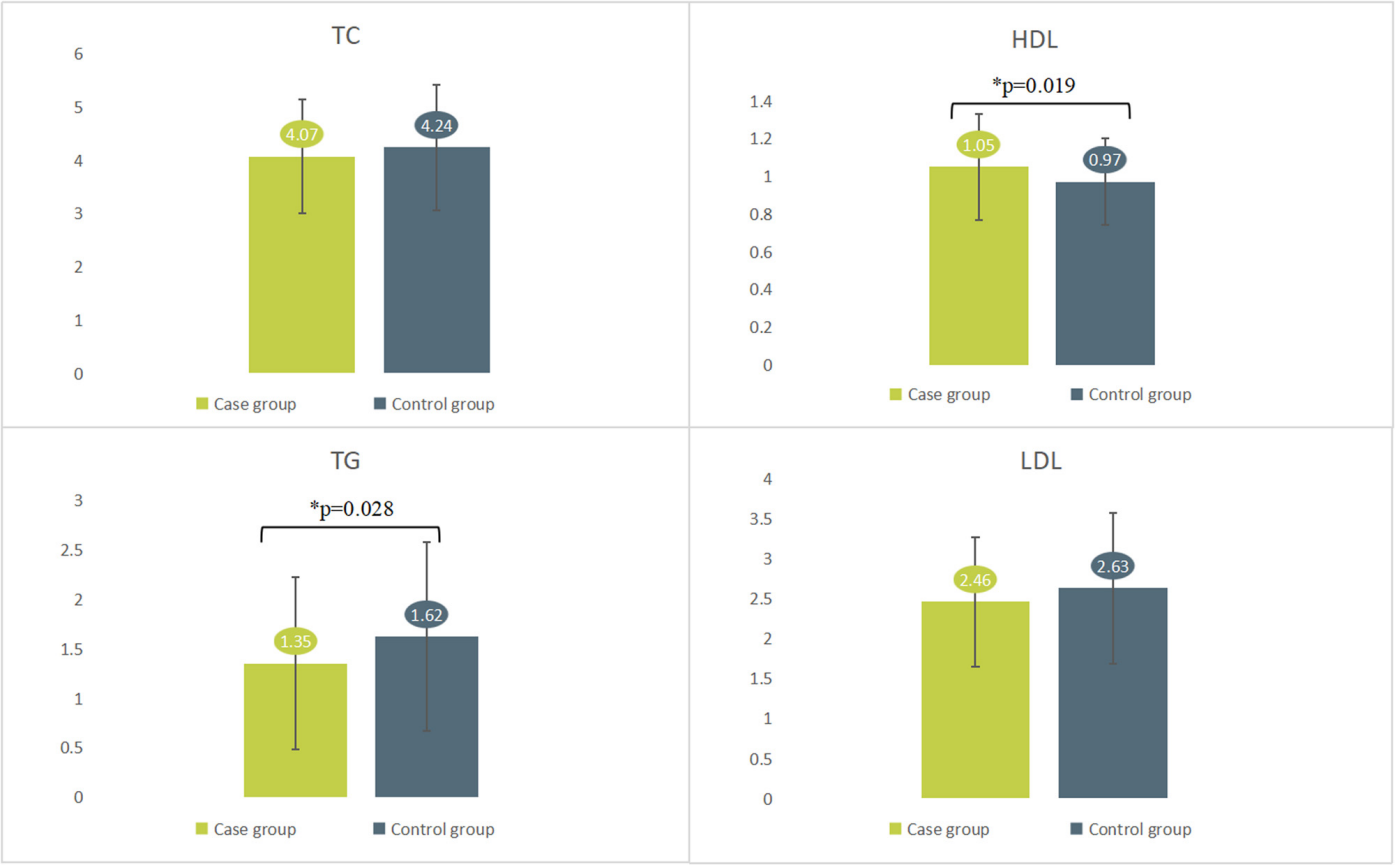
Comparative analysis results of lipid metabolism indexes.

A total of 223 patients were evaluated in this study. The comparative analysis results of demographic information and clinical characteristics are shown in Table 1. There are no significant differences in gender, HBP, diabetes, TP, globulin, ALB, ALT, AST, TC, LDL, UA, BUN, creatinine, Mb, troponin, CK-MB, Hbalc, and blood glucose between the two groups. However, the proportion with fatigue symptom in case group was significantly higher than that of control group (*p* = 0.006). The differences of age, HDL, and TG between case group and control group were statistically significant (*p* = 0.000, *p* = 0.019, and *p* = 0.028). The comparative analysis results of lipid metabolism indexes are shown in Figure 1.

**Table 1:** Demographic information and clinical characteristics of the participants

	Case group (*n* = 90)	Control group (*n* = 133)	*F*/*χ* ^2^	*p*
Gender				
Male	65(72.22%)	110(82.71%)	3.493	0.062
Female	25(27.78%)	23(17.29%)		
Age*	73.04 ± 10.22	64.11 ± 9.27	45.86	0.000
HBP	61(67.78%)	82(61.65%)	0.875	0.350
Diabetes	23(25.56%)	33(24.81%)	0.016	0.900
Fatigue*	21(23.33%)	13(9.77%)	7.637	0.006
TP (total protein)	64.51 ± 5.68	64.24 ± 5.39	0.130	0.719
Globulin	28.01 ± 4.77	26.98 ± 3.75	3.220	0.074
ALB (albumin)	36.5 ± 3.40	37.26 ± 3.24	2.800	0.096
ALT	32.91 ± 49.20	34.21 ± 25.38	0.070	0.797
AST	73.09 ± 108.85	88.47 ± 114.65	1.010	0.317
TC (total cholesterol)	4.07 ± 1.07	4.24 ± 1.18	1.200	0.274
HDL*	1.05 ± 0.28	0.97 ± 0.23	5.560	0.019
LDL	2.46 ± 0.81	2.63 ± 0.94	1.860	0.174
TG (triglyceride)*	1.35 ± 0.87	1.62 ± 0.95	4.920	0.028
UA (uric acid)	348.92 ± 107.93	359.24 ± 100.91	0.530	0.467
Creatinine	83.83 ± 24.78	84.10 ± 36.93	0.000	0.952
BUN (blood urea nitrogen)	6.29 ± 2.72	5.79 ± 2.19	2.270	0.133
Mb (myohemoglobin)	113.54 ± 180.41	86.92 ± 134.05	1.480	0.225
Troponin	7.51 ± 15.47	7.19 ± 13.57	0.020	0.877
CK-MB	67.14 ± 110.42	65.35 ± 104.86	0.010	0.907
Hba1c	132.16 ± 18.29	137.03 ± 17.84	3.840	0.051
Blood glucose	6.82 ± 2.60	6.42 ± 2.31	1.480	0.225

^*^The difference was statistically significant.

### 3.2 Sleep quality and behavior characteristics

The comparative analysis of sleep quality and behavior characteristics between the two groups is shown in Table 2. The ratio of smoking and alcohol consumption in case group is lower than that of control group, but the differences were not statistically significant (*p* = 0.104 and *p* = 0.509). It is worth noting that the sleep status between the two groups is obviously different, the ratio of poor sleep quality in case group was 18.89%, which was significantly higher than 8.27% of control group (*p* = 0.037).

**Table 2:** Comparative analysis of sleep quality and behavior characteristics

	Case group (*n* = 90)	Control group (*n* = 133)	*F*/*χ* ^2^	*p*
Sleep quality*				
Good	71 (78.89%)	121 (90.98%)	6.594	0.037
Average	2 (2.22%)	1 (0.75%)		
Poor	17 (18.89%)	11 (8.27%)		
Smoke				
Yes	5 (5.56%)	16 (12.03%)	2.638	0.104
No	85 (94.44%)	117 (87.97%)		
Alcohol				
Yes	3 (3.45%)	7 (5.26%)	0.436	0.509
No	87 (96.67%)	126 (94.74%)		

^*^The difference was statistically significant.

### 3.3 Analysis of influence factors for CI in CAD patients

Logistic regression analysis was performed to identify the influence factors for CI. Potential influencing factors, with *p* < 0.1 in the univariate analysis, were incorporated into the analytical equation and the results are shown in Table 3. After controlling the possible confounding factors, it was revealed that age was significantly associated with the increased risk of CI in CAD patients (OR = 1.086, 95% CI: 1.047–1.127, *p* = 0.000). Similarly, fatigue symptom was also a risk factor related to CI and the difference was statistically significant (OR = 2.537, 95% CI: 1.068–6.026, *p* = 0.035). Participants with sleep disorder could affect cognitive function, and it was found that sleep quality was significantly relevant to CI (OR = 1.760, 95% CI: 1.106–2.799, *p* = 0.017). However, gender, globulin, ALB, HDL, TG, and Hba1c were not correlated with the risk of CI in CAD patients.

**Table 3:** Identification of influence factors for CI in CAD patients

	OR	95% CI	*p*
Age*	1.086	1.047, 1.127	0.000
Gender	1.100	0.497, 2.432	0.814
Fatigue*	2.537	1.068, 6.026	0.035
Globulin	1.013	0.937, 1.095	0.742
ALB (albumin)	1.040	0.933, 1.160	0.481
Sleep quality*	1.760	1.106, 2.799	0.017
HDL	1.568	0.436, 5.644	0.491
TG	0.784	0.528, 1.163	0.226
Hba1c	0.997	0.978, 1.016	0.741

^*^The difference was statistically significant.

## 4 Discussion

This retrospective case–control study assessed the associations of potentially modifiable cardiovascular risk factors with CI in the patients with CAD. Previous studies have demonstrated that growth of age is strongly linked to CI in the patients with atherosclerotic disease or cardiovascular disorders [19,20]. The mechanism of age-induced CI is a complex process involving multiple factors and the focusing mechanisms mainly include neurobiological factors, physiological factors, and environmental factors [21,22]. The current research indicates that with age, brain structure changes, neurons, and neurotransmitters decrease may directly affect the brain’s information processing and cognitive abilities [21,23]. Studies have also confirmed that physiological changes such as slower blood circulation and lower metabolic rate could lead to insufficient blood supply to the brain, which in turn affects cognitive function [24,25]. Environmental factors such as poor lifestyle habits can accelerate the aging process of the brain, thus affecting cognitive function [26]. In addition, related articles reviewed the role of ncRNA in the pathogenesis of age-related vascular CI and the potential use of ncRNA as diagnostic/prognostic biomarkers [27]. The evidence suggests that age is strongly associated with CI and our research results were consistent with these articles, age was an influence factor for CI in CAD patients.

Increasingly studies on CI have focused on fatigue symptom and its dynamic mechanisms. Fatigue might be a susceptibility indicator to the pernicious effects of stress in individuals at the risk of CI and it is a critical control point to slow down the progression to CI in CAD patients [28,29]. The possible mechanism is that long-term mental stress can lead to physical fatigue, and the overexcitation of the sympathetic nerve, which can cause mood disorders such as anxiety or nervousness, and eventually result in memory loss and CI [30,31]. It is found in this research that fatigue was the risk factor associated with CI in CAD population, which is consistent with previous research studies.

Sleep is a complex physiological process related to psychological and physical health, and it can accelerate or deaccelerate the process of CI [32,33]. Some studies indicate that there is a positive association between cognitive decline and poor sleep quality in chronic disease population [34–36]. Sleep is a reversible risk factor, and early identification and intervention could minimize the impact of CI [32]. Our results are similar to those observations on CI and suggest that sleep quality could be important to the pathological process of CI. At present, cell loss in the forebrain is the possible mechanism of poor sleep quality affecting CI according to the results of experimental investigation [37]. However, some other studies suggest that long-term sleep disorders may lead to neurotransmitter imbalance and cerebral cortex dysfunction, which affects information processing and memory storage, causing problems such as inattention and slow thinking [38]. In addition, decreased cerebral blood flow is another important mechanism of CI. And the abnormal circadian blood pressure rhythm caused by poor sleep quality is closely related to CI [39]. Therefore, prospective studies are needed to explore the mechanisms between potentially modifiable cardiovascular risk factors and CI.

Our major findings suggested that age, fatigue, and sleep quality were the risk factors associated with CI in CAD population. These research results could provide evidence for controlling the progress of CI and improving the life quality of CAD patients. Meanwhile, there were some limitations existing in this retrospective case–control analysis. First, this study was a single center research and the sample size was small, only 223 patients fulfilled inclusion criteria, which would lead to high occurrence of accidental consequences. Prospective, multicenter, large-scale trials were needed to verify these findings. Second, this article was a retrospective analysis, the research data were collected from patient’s report and electronic medical record system, information bias and recall bias were unavoidable. These effects were minimized by comprehensive application of strengthening interview techniques, considering individual differences and background information, multiple assessments and tracking, appropriate statistical methods, and quality control of data. These measures could provide us more accurate and reliable results.

Addressing CI in clinical practice would benefit for personalized treatment programs, interdisciplinary collaboration, application of technological innovations, social support, and rehabilitation services. Through the implementation of these measures, we can provide patients with more comprehensive and effective treatment supports to promote their recovery and return to normal life.

## 5 Conclusion

This retrospective case–control analysis suggested that sleep quality, fatigue, and age were associated with the increased susceptibility of CI in CAD patients. Both CI state and its related factors were involved in the pathological process of CAD. It was revealed that early identification of direct and indirect potential risk factors for CAD makes an important contribution in the treatment and prognosis of CAD.
